# Caring beyond the operating field: social determinants in surgical Nursing

**DOI:** 10.15649/cuidarte.5663

**Published:** 2026-08-25

**Authors:** José Erivelton de Souza Maciel Ferreira, Larissa Rodrigues Siqueira, Aneline Mesquita Sales Carpino, Francisco Edilson Andrade Almeida Júnior, Rian Brito Teles, Marcelo Bruno Almeida Rebouças

**Affiliations:** 1 Nurse, PhD candidate in Nursing at UNILAB, Employee of SESA and the Municipal Sec-retariat of Caucaia, Assistant Manager of the Municipal Hospital of Caucaia, Ceará, Brazil. E-mail: eriveltonsmf@gmail.com Nurse, PhD candidate in Nursing at UNILAB Ceará Brazil eriveltonsmf@gmail.com; 2 Doctor of Nursing from UFC (Federal University of Ceará), employee of SESA and the Municipal Secretariat of Caucaia, Ceará, Brazil. E-mail: larissars1994@gmail.com Doctor of Nursing from UFC (Federal University of Ceará) Ceará Brazil larissars1994@gmail.com; 3 Nurse graduated from UNIFATENE, Postgraduate in Quality Management in Health, Coordinator of the Quality Department of the Municipal Hospital of Caucaia, Ceará, Brazil. E-mail: anelinesalles@hotmail.com Nurse graduated from UNIFATENE Ceará Brazil anelinesalles@hotmail.com; 4 Nurse graduated from UNIFATENE, Administrative Director of the Municipal Hospital of Caucaia, Ceará, Brazil. E-mail: edilsonaaj@gmail.com Nurse graduated from UNIFATENE Ceará Brazil edilsonaaj@gmail.com; 5 Medical Doctor from Unichristus, Medical residency in Internal Medicine and specializa-tion in Internal Medicine, General Director of the Municipal Hospital of Caucaia, Ceará, Brazil. E-mail: rianteles935@gmail.com Medical Doctor from Unichristus Ceará Brazil rianteles935@gmail.com; 6 Nurse graduated from the Catholic University Rainha do Sertão, Assistant Coordinator at the São Vicente Institute, Caucaia, Ceará, Brazil. E-mail: marceloreboucas16@gmail.com Nurse graduated from the Catholic University Rainha do Sertão Ceará Brazil marceloreboucas16@gmail.com

**Keywords:** Social Determinants of Health, Perioperative Nursing, Perioperative Care, Health Equity, Determinantes Sociales de la Salud, Enfermería Perioperatoria, Atención Perioperativa, Equidad en Salud, Determinantes Sociais da Saúde, Enfermagem Perioperatória, Assistência Perioperatória, Equidade em Saúde

## Abstract

**Introduction:**

In the surgical setting, social determinants of health directly influence surgical outcomes, from indication to postoperative recovery, reflecting social inequalities that require critical nursing attention.

**Objective:**

To reflect, in light of a narrative review, on the influence of social determinants of health on surgical outcomes and their implications for perioperative Nursing.

**Content Synthesis:**

This is a theoretical-reflective essay grounded in a structured narrative review conducted in the PubMed database between April and June 2025. Twenty-six articles were included. The analysis was carried out through an integrative thematic synthesis organized according to the Dahlgren and Whitehead model. The findings indicate that incorporating social determinants broadens the scope of nursing practice across different levels. At the macro level, it involves political and institutional engagement, participation in policy formulation, and equitable resource management. Regarding mesodeterminants, expanded social assessment, individualized care planning, articulation with community networks, and culturally sensitive health education stand out. At the micro level, individualized clinical assessment, psychosocial support, humanized care, postoperative planning, and the use of technologies to support continuity of care are included. This approach strengthens comprehensiveness and social justice within the surgical context.

**Conclusion:**

Integrating social determinants of health into Nursing practice in the surgical setting strengthens patient safety, improves quality of care, and contributes to reducing health inequities.

## Resumen

**Introducción:**

En el quirófano, los determinantes sociales de la salud influyen directamente en los resultados quirúrgicos, desde la indicación hasta el periodo postoperatorio, reflejando desigualdades sociales que requieren una atención prioritaria por parte de la enfermería.

**Objetivo:**

Reflexionar, mediante una revisión narrativa, sobre la influencia de los determinantes sociales de la salud en los resultados quirúrgicos y sus implicaciones para la enfermería perioperatoria.

**Síntesis de Contenido:**

Este es un ensayo teórico-reflexivo basado en una revisión narrativa estructurada realizada en la base de datos PubMed entre abril y junio de 2025. Se incluyeron veintiséis artículos. El análisis se realizó mediante una síntesis temática integradora, organizada según el modelo de Dahlgren y Whitehead. Se evidenció que la incorporación de los determinantes sociales amplía el alcance de la práctica de enfermería en diferentes niveles. A nivel macro, implica el compromiso político-institucional, la participación en la formulación de políticas y la gestión equitativa de los recursos. En cuanto a los determinantes sociales de la salud, los aspectos clave incluyen la valoración social ampliada, la planificación de cuidados individualizados, la articulación con redes comunitarias y la educación sanitaria culturalmente sensible. Los determinantes microeconómicos incluyen la valoración clínica individualizada, el apoyo psicosocial, la atención humanizada, la planificación postoperatoria y el uso de tecnologías para la continuidad asistencial. Este enfoque fortalece la integralidad y la justicia social en el contexto quirúrgico.

**Conclusiones:**

La integración de los determinantes sociales de la salud en la práctica de enfermería en el centro quirúrgico fortalece la seguridad del paciente, mejora la calidad de la atención y contribuye a reducir las inequidades en salud.

## Resumo

**Introdução:**

No centro cirúrgico, os determinantes sociais da saúde influenciam diretamente os desfechos cirúrgicos, da indicação ao pós-operatório, refletindo desigualdades sociais que exigem atenção crítica da enfermagem.

**Objetivo:**

Refletir à luz de uma revisão narrativa sobre a influência dos determinantes sociais da saúde nos desfechos cirúrgicos e suas implicações para a Enfermagem perioperatória.

**Síntese do conteúdo:**

Trata-se de um ensaio teórico-reflexivo fundamentado em revisão narrativa estruturada realizada na base PubMed entre abril e junho de 2025. Foram incluídos 26 artigos. A análise ocorreu por síntese temática integradora, organizada a partir do modelo de Dahlgren e Whitehead. Evidenciou-se que a incorporação dos determinantes sociais amplia o escopo da prática de enfermagem em diferentes níveis. No plano macro, envolve engajamento político-institucional, participação na formulação de políticas e gestão equitativa de recursos. Nos mesodeterminantes, destaca-se a avaliação social ampliada, o planejamento assistencial individualizado, a articulação com redes comunitárias e a educação em saúde culturalmente sensível. Nos microdeterminantes, incluem-se avaliação clínica individualizada, suporte psicossocial, acolhimento humanizado, planejamento pós-operatório e uso de tecnologias para continuidade do cuidado. Essa abordagem fortalece a integralidade e a justiça social no contexto cirúrgico.

**Conclusões:**

A integração dos determinantes sociais da saúde à prática da Enfermagem no centro cirúrgico fortalece a segurança do paciente, aprimora a qualidade do cuidado e contribui para a redução de iniquidades em saúde.

## Introduction

Human health is influenced by multiple factors that extend beyond biological processes and are embedded within social, economic, cultural, and environmental structures. Social Determinants of Health (SDH), defined as the conditions in which people are born, grow, live, work, and age, directly influence patterns of health, illness, and recovery within populations^1^. From a social justice perspective, these determinants are not merely contextual variables but structural forces that generate systemic inequalities and shape therapeutic trajectories and clinical outcomes. In hospital settings, particularly within surgical centers, understanding these determinants is essential for promoting ethical, comprehensive, and person-centered care^2^.

To examine this complex network of influences, the framework proposed by Dahlgren and Whitehead is widely used. This model categorizes SDH into macro-determinants (structural factors such as public policies, economic conditions, and social protection systems), meso-determinants (intermediate factors including housing conditions, work environments, access to healthcare services, and social support networks), and micro-determinants (individual and behavioral factors)^2^. By illustrating the dynamic interactions between social structures and individual experiences, the model highlights how health inequalities emerge and persist across populations. Consequently, these determinants influence surgical outcomes throughout the entire continuum of care, from surgical indication to the postoperative period^3^,^4^.

Recent evidence has demonstrated that factors such as income, health insurance coverage, educational attainment, food security, housing conditions, community context, and the physical environment are associated with significant differences in prognosis, length of hospital stay, quality of life, and postoperative complications^5^,^6^. EStudies have shown that the absence of private health insurance, low household income, and residence in socially vulnerable areas are associated with increased risks of adverse postoperative outcomes, including infections, hospital readmissions, and postoperative delirium among older adults^5^,^6^. These findings suggest that surgical risk extends beyond traditional biomedical factors and encompasses social determinants that influence healthcare access, delivery, and outcomes.

Addressing Social Determinants of Health requires a multidimensional approach capable of capturing the complexity of these interconnected factors^7^. The stratification of clinical data according to different SDH domains, as recommended by international guidelines, facilitates the identification of health disparities and supports the development of targeted interventions, including referrals to social services, engagement of community health workers, and adaptations to perioperative care plans^1^,^7^,^8^. Despite these recommendations being well established in the literature, their implementation in routine surgical practice remains limited.

Although healthcare professionals increasingly recognize the importance of SDH, their systematic assessment, documentation, and integration into clinical workflows remain insufficient. Barriers such as the absence of standardized protocols, inadequate professional training, and limited availability of screening instruments hinder the incorporation of SDH into everyday clinical practice^9^. Consequently, a substantial gap persists between scientific knowledge and its practical application, particularly in complex hospital environments such as surgical centers. This gap represents not only an operational challenge but also an ethical and epistemological concern, reflecting the difficulty of integrating critical perspectives on social inequities into the predominantly technocratic model of perioperative care.

Within this context, perioperative nursing plays a pivotal role in integrating technical and scientific expertise with a strong ethical commitment to health equity through the lens of Social Determinants of Health. By recognizing these determinants as integral components of patient care, nurses can adopt a critical and proactive approach that extends beyond the performance of clinical procedures to encompass reflection, advocacy, and social transformation^10^,^11^. Grounded in Emancipatory Nursing Theories, this perspective requires critical awareness of the power structures that shape health and healthcare delivery, as well as an active commitment to reducing inequities. It also demands sensitivity to identify hidden vulnerabilities, dedication to social justice, and the competence to implement interventions that address the individual throughout the surgical journey^11^.

Therefore, integrating Social Determinants of Health into perioperative clinical practice should not be viewed merely as a technical recommendation but as an ethical imperative for contemporary nursing. Such integration is consistent with a praxis committed to comprehensive care and the improvement of population health conditions. Accordingly, this article aims to reflect, through a narrative review, on the influence of Social Determinants of Health on surgical outcomes and their implications for perioperative nursing practice.

## Content Summary

This study is a theoretical-reflective essay based on a narrative literature review conducted in the PubMed database between April and June 2025. This methodological approach was selected because of its analytical and interpretative nature, which is consistent with the study objective of critically examining the relationship between Social Determinants of Health (SDH) and surgical outcomes, with particular emphasis on implications for perioperative nursing practice^12^.

Unlike systematic or scoping reviews, narrative reviews do not seek to provide an exhaustive synthesis or quantitative assessment of the available evidence. Instead, they aim to develop an interpretative understanding by integrating diverse theoretical and empirical perspectives on a given topic^13^.

The focus of this study was to explore how Social Determinants of Health influence surgical care and perioperative nursing practice. A narrative literature review was chosen because its purpose is not to identify all available evidence systematically, but rather to develop a comprehensive and contextualized understanding of the phenomenon under investigation.

The review was conducted exclusively in the PubMed database due to its international relevance, broad coverage of health-related research, and extensive indexing of peer-reviewed scientific journals. This database was considered appropriate for providing a comprehensive overview of surgical care and its relationship with Social Determinants of Health at both national and international levels.

The search strategy employed the descriptors “Social Determinants of Health,” “Surgical Care,” “Nursing Practice,” and “Health Disparities,” combined using Boolean operators (AND/OR). The search was restricted to studies published within the previous five years. Given the reflective nature of this narrative review, methodological procedures typically associated with systematic, integrative, or scoping reviews were not applied. Instead, emphasis was placed on the critical and integrative interpretation of the literature, with the aim of exploring the relationships among Social Determinants of Health, access to surgical care, surgical outcomes, and the role of nursing within this context.

Although the selected studies did not always focus directly on nursing practice, the analysis was guided by a critical-interpretative perspective that integrated recent scientific evidence with the authors’ professional experience in surgical and perioperative care^12^,^14^.

The analysis was conducted in three stages: (1) comprehensive reading of the selected articles and extraction of findings related to Social Determinants of Health and surgical outcomes; (2) identification of thematic patterns and conceptual convergences; and (3) organization of the findings into three analytical dimensions: structural macro-determinants, institutional meso-determinants, and individual micro-determinants, according to the framework proposed by Dahlgren and Whitehead^15^.

A total of 26 articles published predominantly between 2021 and 2025 were included, together with seminal theoretical references considered essential for the conceptual foundation of the study^16^-^41^. Most publications consisted of retrospective and cross-sectional observational studies based on population databases, as well as systematic reviews, narrative reviews, and qualitative studies. Most of research was conducted in high-income countries, particularly the United States and European nations, and addressed specialties such as oncology, colorectal surgery, thoracic surgery, orthopedics, and neurosurgery. The principal outcomes investigated included postoperative complications, hospital readmissions, length of hospital stay, stage at diagnosis, and access to surgical treatment, frequently examined in relation to socioeconomic indicators such as income, health insurance status, and social vulnerability.

**Figure 1:**
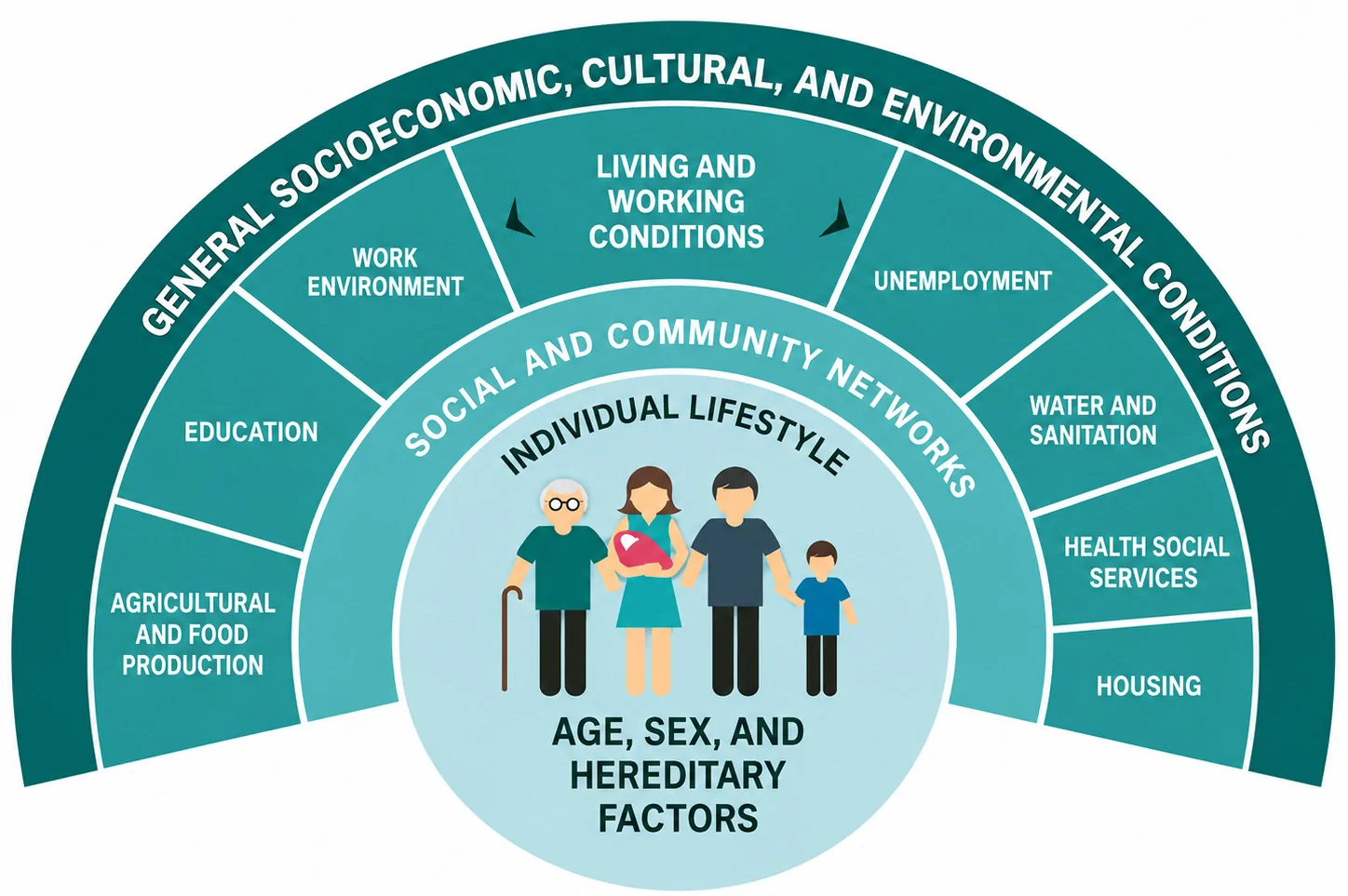
Social determinants of health

*Note: Adapted from Dahlgren and Whitehead -2021^15^*

Before presenting the reflective analysis, it is important to revisit the framework proposed by Dahlgren e Whitehead^15^, which conceptualizes Social Determinants of Health as interconnected and interdependent layers. Based on this model, the present study organized its reflections into three analytical dimensions corresponding to the interactions among structural factors, institutional mediators, and individual conditions within the surgical context Figure 1.

Based on this analytical framework and the reflections developed throughout the study, a graphical model was created using Bizagi Modeler software with the aim of visually illustrating the relationships between the different levels of Social Determinants of Health and potential areas for intervention in surgical nursing practice. This graphical representation does not constitute an empirical finding; rather, it serves as a didactic and analytical tool designed to synthesize the thematic framework developed from the literature.

**Macro-determinants of health and the emancipatory practice of Nursing in surgical care**

Macro-determinants of health constitute the broadest layer of the framework proposed by Dahlgren and Whitehead, encompassing the social, economic, political, cultural, and environmental factors that structurally shape patterns of illness, access to healthcare services, and recovery trajectories within populations^15^. The studies analyzed consistently demonstrate associations between these determinants and disparities in access to surgical treatment, stage of disease presentation, and the occurrence of postoperative complications. Therefore, within the context of Surgical Nursing, understanding these determinants is essential for identifying the systemic origins of health inequities and guiding a critical practice that extends beyond technical care to promote social justice, equity, and citizenship within healthcare settings.

From a critical perspective, macro-determinants are not external influences on care but structural forces that shape the daily functioning of surgical services, affecting service organization, patient profiles, resource allocation, and the practical possibilities for nursing action^16^,^17^. These determinants are reflected, for example, in public healthcare financing policies, which directly influence hospital capacity and the availability of elective and emergency surgical procedures. In Brazil, although the Unified Health System (SUS) is guided by the principles of universality and equity, resource constraints often limit its ability to meet growing healthcare demands, particularly in medium- and high-complexity services. As a result, long waiting lists, procedure cancellations, and shortages of essential supplies remain common, especially in socioeconomically disadvantaged regions^5^,^18^.

This reality is also shaped by broader political and economic structures. The unequal distribution of public investment, combined with persistent socioeconomic inequalities, contributes to inadequate hospital infrastructure, insufficient operating rooms, limited postoperative bed availability, and shortages of essential materials and medications^19^. The literature reviewed here associates these structural deficiencies with delays in treatment and increased risks of adverse outcomes among vulnerable populations. Such conditions compromise not only patient safety but also the dignity of care, an ethical dimension that nursing practice cannot overlook.

Furthermore, prevailing socioeconomic inequalities characterized by income concentration, labor market insecurity, and racial and territorial disparities create substantial barriers to surgical access. Socioeconomic conditions influence patients’ ability to complete preoperative assessments, attend healthcare appointments, maintain postoperative follow-up, and adhere to treatment recommendations^20^. These findings reinforce the notion that surgical risk is not solely determined by clinical factors but is deeply embedded within structural conditions that shape healthcare experiences and outcomes.

Within this context, the role of Surgical Nursing can be understood as both caring and transformative. While actively participating in direct patient care, nurses must also act as agents of social and institutional change, integrating technical expertise with an ethical commitment to addressing health inequities. Recognizing the effects of macro-determinants, including structural racism, territorial inequalities, institutional violence, and the social exclusion of marginalized populations, allows nurses to interpret delays in treatment, complications, and treatment discontinuation not as individual failures but as manifestations of historically produced social inequities^3^,^21^.

In this regard, emancipatory nursing praxis offers both an analytical framework and a practical response to these structural challenges. Drawing on the work of Peart and MacKinnon^10^, and Velasco and Reed^11^, this perspective emphasizes critical awareness, ethical responsibility, and transformative action in response to systems of domination and exclusion. Within surgical settings, this requires challenging the normalization of inequalities, advocating for vulnerable patients, and collectively developing care strategies that incorporate Social Determinants of Health into routine clinical practice.

This broader perspective expands the possibilities for nursing action at the macro-determinant level through three key dimensions:

• Political engagement in institutional decision-making: nurses in leadership positions can advocate for structural improvements in surgical services, including workforce expansion, infrastructure development, and the acquisition of technologies. By utilizing epidemiological evidence and social indicators, nurses can support equitable resource allocation and promote distributive justice within healthcare institutions^9^.

• Participation in health policy development: Engagement in health councils, advisory boards, and policy forums enables nurses to contribute to the formulation of policies aimed at expanding surgical access for historically marginalized populations, including Indigenous peoples, quilombola communities, and individuals experiencing homelessness^22^. Such participation strengthens the social role of nursing and its capacity to influence the structural determinants of health.

• Equitable management of healthcare resources: Through surgical scheduling, workflow organization, and care supervision, nurses can help ensure that administrative decisions consider the needs of socially vulnerable populations. This may include implementing protocols that incorporate social vulnerability screening tools, Social Vulnerability Indices (SVI), and mechanisms for identifying barriers to healthcare access^4^,^23^.

Therefore, the analysis of macro-determinants of health within the surgical context is not merely a theoretical exercise but a practical necessity for healthcare professionals committed to equity and social justice. Nursing practice must be grounded in a broader understanding of health as a fundamental human right and supported by critical, interdisciplinary professional education that is responsive to the sociopolitical dynamics shaping healthcare systems^7^.

**Meso-determinants of health and their influence on surgical care**

Within the framework proposed by Dahlgren and Whitehead^15^, meso-determinants of health occupy the intermediate layer between macro- and micro-determinants, encompassing institutional, organizational, territorial, and community factors that mediate the effects of structural determinants on individuals’ experiences within healthcare systems. In the context of Surgical Nursing, these factors are critical to the quality, safety, and continuity of care, serving as key mediators between structural inequities and clinical outcomes^3^,^4^.

Unlike macro-determinants, which relate to the broader organization of society, meso-determinants are expressed through the functioning of healthcare institutions, service management practices, the integration of care networks, and the capacity for collaboration among healthcare professionals and patients. These dimensions influence the entire surgical pathway, from the indication of surgery to hospital discharge and postoperative follow-up, directly affecting treatment adherence, complication rates, and equitable access to care^23^,^24^. Furthermore, the studies included in this review indicate that poor communication between levels of care and inadequate transitional care planning contribute to preventable hospital readmissions. Within this context, nursing not only accompanies patients throughout the surgical process but also serves as a fundamental source of support, guidance, and humanized care.

Among the meso-determinants most relevant to surgical care are institutional workflow organization, operating room and bed management, communication across levels of care, transportation availability, community support systems, and multidisciplinary team integration^3^. Barriers such as surgery cancellations due to incomplete preoperative testing, hospital discharge without continuity-of-care planning, and the absence of social assessments during hospitalization reflect systemic deficiencies that compromise patient safety and surgical outcomes^9^. These challenges have been reported across diverse healthcare settings, particularly in resource-constrained environments.

In Brazil, such barriers are further exacerbated by regional disparities and the historical fragmentation of the Unified Health System (SUS). In regions with limited access to specialized healthcare services, including rural areas and urban peripheries, patients often face substantial obstacles to obtaining timely and safe surgical care. Moreover, postoperative follow-up is frequently compromised by inadequate coordination between hospitals and primary healthcare services^25^. These gaps represent critical meso-determinants that intensify health inequities among socioeconomically vulnerable populations.

Because nurses are involved in every stage of the surgical journey, Surgical Nursing occupies a strategic position in addressing meso-determinants of health. Nurses can identify hidden social vulnerabilities, implement educational interventions tailored to patients’ realities, coordinate formal and informal support networks, and facilitate access to institutional and community resources. Such actions require reflective practice, sensitivity to social and territorial contexts, and a strong commitment to equity from the initial patient assessment onward^22^,^26^. Evidence suggests that coordinated, person-centered interventions are associated with improved treatment adherence and reduced risks of discontinuity in surgical care^26^,^27^.

For this mediation role to be effective, healthcare institutions must recognize the importance of incorporating social assessment into surgical planning and establish structures that support intersectoral collaboration. The absence of protocols that integrate social factors into surgical risk assessment, as well as the undervaluation of transitional care practices, limits nursing’s ability to address meso-determinants effectively and perpetuates avoidable gaps in care delivery^3^,^9^.

In addition, cultural competence and effective communication are essential tools for responding to the sociocultural diversity of surgical patients. A person-centered approach that respects individuals’ beliefs, language, lived experiences, and community ties strengthens the therapeutic relationship and promotes patient engagement throughout recovery^24^. Health education, when adapted to patients’ cultural and social contexts, becomes a powerful strategy for enhancing autonomy and reducing postoperative risks.

Therefore, a critical nursing approach to meso-determinants requires strengthening interdisciplinary collaboration, integrating social assessment tools into clinical practice, and developing collaborative networks among healthcare services, community organizations, and social support systems. Such an approach is essential to ensure that surgical care extends beyond technical procedures and is delivered as a comprehensive, socially responsive, and equity-oriented practice. As demonstrated by several studies included in this review, institutional strategies that promote care coordination and intersectoral communication play an important role in mitigating inequities along the surgical pathway^24^-^28^.

Key nursing interventions addressing meso-determinants of health in the surgical context include:

• Comprehensive social assessment and individualized care planning: nurses should conduct thorough assessments upon admission and throughout hospitalization to identify social barriers to recovery, including inadequate housing conditions, food insecurity, lack of caregiver support, and transportation difficulties. Based on these assessments, individualized care plans can be developed, incorporating referrals to social services and coordination with primary healthcare providers^3^.

• Community and territorial engagement: strengthening connections with primary healthcare services and formal or informal community support networks, such as community health centers, Social Assistance Reference Centers (CRAS), religious organizations, and local associations, can help establish support systems for surgical patients after discharge. Such integration reduces care fragmentation, prevents readmissions related to treatment discontinuation, and promotes continuity of care within vulnerable communities^22^. Effective intersectoral and interprofessional communication is essential to achieving these goals. Strategies such as shared care meetings, streamlined referral pathways, integrated care protocols, and shared electronic health records can facilitate communication across levels of care and strengthen healthcare continuity^9^,^26^.

• Culturally sensitive health education: preoperative and postoperative education should be tailored to patients’ sociocultural contexts and levels of health literacy. The use of plain language, visual educational materials, and family involvement can improve understanding of treatment recommendations and enhance adherence to care plans^24^.

Meso-determinants of health therefore represent a crucial dimension of Surgical Nursing practice, requiring professionals who are prepared to understand patients’social realities and proactively address institutional and community barriers to care. Integrating this perspective into clinical practice not only improves surgical outcomes but also reinforces the role of nursing as an agent of social transformation within healthcare systems.

**The practice of micro-determinants in surgical nursing**

The role of Surgical Nursing in addressing micro-determinants of health represents a concrete expression of praxis as an ethical, reflective, and transformative form of action. Located at the innermost layer of the Dahlgren and Whitehead^15^ model, micro-determinants encompass individual and behavioral factors that directly influence the health–disease process, including lifestyle, health literacy, emotional well-being, family support, treatment adherence, and self-care practices. In surgical settings, these factors assume particular importance because they influence both preoperative preparation and postoperative recovery, ultimately affecting clinical outcomes and the quality of care provided^27^,^28^.

From the perspective of critical praxis, the surgical nurse moves beyond the role of a technical executor of clinical protocols and becomes an active participant in the development of individualized and humanized care. This approach requires attentive listening, a comprehensive understanding of each patient's circumstances, and the ability to implement interventions that extend beyond conventional clinical practice. Preoperative anxiety, for example, may significantly affect perceived pain, immune function, and wound healing, requiring nurses to adopt supportive and patient-centered approaches that incorporate emotional support and educational strategies tailored to patients’ sociocultural contexts^29^.

Numerous other micro-determinants may influence surgical experience, including fear of anesthesia, limited understanding of the surgical procedure, low health literacy, and the absence of social support networks. In all these situations, nursing practice must remain critically engaged and responsive, recognizing and addressing the subjective and contextual factors that shape surgical care.

When discussing care practices in surgical settings, an educational, dialogical, and culturally sensitive approach is essential. Such praxis acknowledges patients’ individual characteristics and promotes autonomy throughout the surgical process. The use of accessible language, visual educational materials, and participatory teaching strategies can enhance understanding of wound care, warning signs, pain management, and medication adherence^10^. This educational dimension not only reduces postoperative risks but also strengthens the therapeutic relationship and promotes patients’ active participation in their own care.

Contemporary healthcare realities demonstrate that many micro-determinants are profoundly influenced by broader social vulnerabilities. Food insecurity, limited access to medications, inadequate family support, and precarious housing conditions can directly affect treatment adherence and the effectiveness of postoperative rehabilitation. In this context, nursing plays a crucial role in identifying barriers and seeking practical solutions tailored to each patient’s circumstances, even in resource-constrained settings^30^,^31^. These factors have been consistently identified in qualitative and observational studies as significant obstacles to surgical recovery, particularly among socially vulnerable populations.

The incorporation of digital health technologies represents an important complementary strategy for addressing micro-determinants of health. Mobile applications for symptom monitoring, medication reminders, telehealth consultations, and educational platforms can facilitate longitudinal follow-up of surgical patients, strengthen self-care behaviors, and reduce the risk of postoperative complications^32^. The use of these technologies may also enhance patient engagement and improve the overall surgical care experience^32^.

Addressing micro-determinants effectively also requires alignment with public policies and healthcare models that prioritize person-centered and comprehensive care. Primary Health Care services play a fundamental role in ensuring continuity of postoperative follow-up, particularly among populations experiencing social vulnerability^33^. However, several studies have highlighted persistent operational challenges in the integration of healthcare services across different levels of care, which may compromise continuity and quality of postoperative management^33^,^34^.

Evidence suggests that nursing interventions focused on micro-determinants are associated with improved treatment adherence and better surgical recovery outcomes. Studies have demonstrated that patients receiving individualized educational interventions delivered by nurses experience lower hospital readmission rates^34^. Furthermore, continuous psychosocial support combined with intersectoral collaboration contributes significantly to improved clinical outcomes among surgical patients living in highly vulnerable social contexts^35^.

Thus, practical examples of nursing interventions addressing micro-determinants of health include the following:

• Individualized assessment and comprehensive monitoring: upon admission for surgery, nurses conduct a thorough assessment focused on comorbidities, lifestyle behaviors, nutritional status, health literacy, and family support. For example, among patients with diabetes, close monitoring of glycemic control and targeted education are essential for preventing complications such as surgical site infections and delayed wound healing^36^. The evidence reviewed reinforces the importance of individualized assessment as a key strategy for preventing avoidable perioperative complications.

• Promotion of personalized health education: surgical nurses provide guidance to patients and their families regarding smoking cessation, physical activity, adequate nutrition, and other health behaviors that may optimize surgical recovery. Educational interventions should be tailored to patients’ cultural backgrounds, cognitive abilities, and health literacy levels to facilitate sustainable behavioral change^37^. Encouraging patients to resume self-care practices from the early postoperative period is an important component of recovery planning.

• Psychosocial support and therapeutic communication: recognizing that fear, anxiety, and emotional distress may negatively affect surgical recovery, nurses establish supportive environments based on trust, empathy, and active listening^38^,^39^. Evidence suggests that structured psychosocial interventions can improve patients’ experiences and strengthen adherence to postoperative care recommendations.

• Planning and coordination of postoperative care: nursing practice is also expressed through the individualized coordination of care according to patients’ clinical, social, and emotional needs. Examples include implementing early mobilization strategies for patients at increased risk of deep vein thrombosis and providing detailed wound-care education for immunocompromised individuals^40^. These interventions illustrate the importance of care management grounded in clinical judgment, evidence-based practice, and contextual awareness, thereby promoting safety, effectiveness, and equity throughout the recovery process.

• Use of digital and assistive technologies: digital health tools, including remote monitoring applications, medication reminder systems, telehealth platforms, and educational resources, can reinforce self-management and continuity of care, particularly among patients with limited access to healthcare services^41^.

Micro-determinants of health therefore constitute a critical dimension of Surgical Nursing practice. Their systematic assessment and incorporation into care planning contribute to improved quality of care, enhanced patient safety, greater treatment adherence, and a reduction in preventable perioperative complications.

**Application of surgical nursing practice considering the Social Determinants of Health (SDH).**

Surgical Nursing practice, when informed by the different levels of the Social Determinants of Health, is expressed through interconnected actions that range from policy advocacy and institutional engagement to individualized, person-centered care. Table 1 presents an analytical synthesis developed from the reviewed literature and the authors’ critical-reflective interpretation, illustrating how nurses can operationalize reflective praxis across each determinant level to promote more equitable, effective, and patient-centered surgical care.

**Table 1:** Nursing practice in the macro, meso and micro determinants of health in the surgical context

Level of determinants	Nursing practice	Expected results
Macro-determinants	Participate in councils advocating for equitable health policies. To propose protocols that optimize the use of hospital resources. To empower teams in social justice and social determinants of health.	To improve access and equity in surgical services. Reduce waiting times in queues and strengthen security in customer service. To broaden the awareness and sensitivity of the multidisciplinary team.
Meso-determinants	Assess social conditions and support network upon hospital admission. Coordinate support services (social assistance, shared transportation). To develop culturally appropriate health education. Involve family members in the care process.	Identify social barriers and facilitate comprehensive care To ensure continuity of care and accessibility. To facilitate understanding and adherence to treatment. To strengthen emotional and practical support for the patient.
Micro-determinants	Assess the patient's lifestyle habits, mental health, and level of knowledge. To carry out individualized educational interventions on self-care. To offer emotional support and psychological referral. Conduct post-discharge follow-up (when available) via telephone contact or follow-up strategies.	Personalize care and identify risks. To promote autonomy and safety in postoperative management. Reduce anxiety and improve well-being. Identify complications early and reinforce adherence.

**Figure 2:**
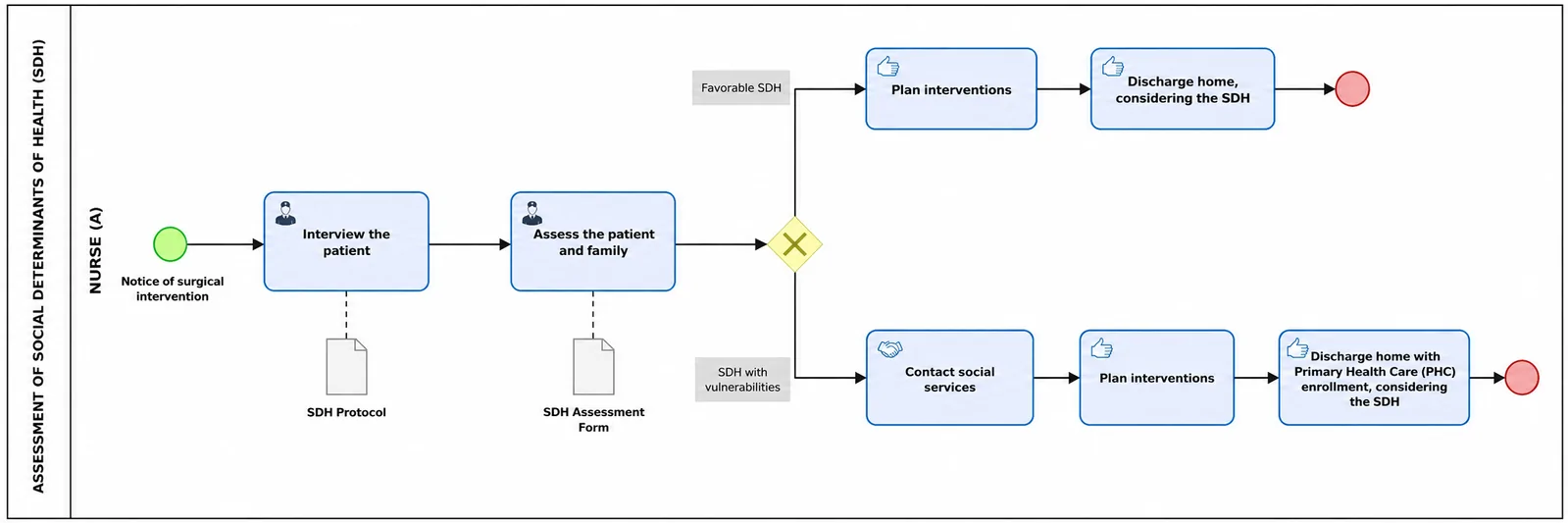
Flowchart for the assessment of DSS in the perioperative context

*Note: This flowchart describes the assessment of the DSS (Discriminative Social Structure) in the perioperative process, summarizing the clinical journey of the surgical patient from the communication of the diagnosis to hospital discharge, highlighting the critical points of nursing intervention guided by the DSS.*

This perspective broadens the understanding of surgical care by integrating structural, institutional, and individual dimensions into the care process, as highlighted in the studies analyzed, thereby reinforcing the profession’s ethical and social commitment to health equity. Furthermore, based on the thematic synthesis developed in this essay, Figure 2 was created as a didactic-analytical framework illustrating the assessment and integration of Social Determinants of Health (SDH) within the perioperative context.

The framework is organized as a logical and chronological sequence, beginning with the communication of the need for surgery, a stage at which factors related to Social Determinants of Health (SDH) may first be identified. The preoperative assessment includes the evaluation of macro-, meso-, and micro-determinants, such as socioeconomic conditions, access to information, and family support. During surgical preparation, nurses may implement educational interventions, provide emotional support, and identify specific clinical and social risks.

The transition through the operating room and the Post-Anesthesia Care Unit (PACU) represents critical stages during which clinical monitoring is reinforced while attention is given to individual vulnerabilities and care needs. The framework culminates in hospital discharge and the patient’s return home, emphasizing the importance of strategies that incorporate Social Determinants of Health into perioperative care to promote continuity of care, treatment adherence, and the prevention of postoperative complications.

Table 1 and Figure 2 therefore synthesize the analytical interpretation developed in this study, highlighting the strategic potential of Surgical Nursing to integrate Social Determinants of Health into perioperative care and thereby contribute to more equitable, comprehensive, and patient-centered healthcare.

## Conclusions

In this context, the literature reviewed demonstrates that Nursing plays a fundamental role in recognizing patients’ individual circumstances and implementing personalized care strategies based on an understanding of the Social Determinants of Health (SDH) that shape their health experiences. Such an approach requires comprehensive assessments that extend beyond biomedical factors to include nutritional status, treatment adherence, psychosocial support, environmental conditions, and preventive measures. By addressing these dimensions, nurses can help minimize perioperative risks and promote more effective recovery following hospital discharge or transition to other healthcare services for continued treatment.

As discussed throughout this essay, nursing interventions across the different layers of the Social Determinants of Health are inherently interconnected and interdependent, requiring coordination among levels of care and integration between the clinical and social dimensions of healthcare. Within surgical settings, the literature reviewed indicates that structural, organizational, and individual factors influence access to surgical procedures, the quality of perioperative care, and clinical outcomes. Recognizing these determinants is therefore essential for addressing health inequities and promoting equitable surgical care.

The systematic incorporation of Social Determinants of Health into clinical practice, together with the development of institutional protocols that acknowledge local realities and population-specific vulnerabilities, may contribute to greater equity in surgical care. The evidence reviewed suggests that personalized educational interventions, intersectoral collaboration, and attention to socioeconomic conditions are associated with improved treatment adherence and better postoperative recovery outcomes.

Finally, the application of the Dahlgren and Whitehead framework to the surgical context provides a valuable analytical perspective for understanding how social determinants influence perioperative care. Incorporating this perspective into nursing practice may strengthen clinical decision-making, enhance patient safety, improve the quality of surgical care, and promote a more comprehensive approach that is responsive to social inequities and committed to health equity.
